# Aptamers as Diagnostic Tools in Cancer

**DOI:** 10.3390/ph11030086

**Published:** 2018-09-11

**Authors:** Dario Ruiz Ciancio, Mauricio R. Vargas, William H. Thiel, Martin A. Bruno, Paloma H. Giangrande, María Belén Mestre

**Affiliations:** 1 Biomedical Science Institute (ICBM), Catholic of Cuyo University, San Juan, CP 5400, Argentina; ruizdarioe@gmail.com (D.R.C.); bioquimicamauro@gmail.com (M.R.V.); martinbruno_investigacion@uccuyo.edu.ar (M.A.B.); 2 Department of Internal Medicine, University of Iowa, Iowa City, IA 52246, USA; william.thiel@gmail.com (W.H.T.); paloma-giangrande@uiowa.edu (P.H.G.)

**Keywords:** aptamer, cancer, diagnosis, imaging

## Abstract

Cancer is the second leading cause of death worldwide. Researchers have been working hard on investigating not only improved therapeutics but also on early detection methods, both critical to increasing treatment efficacy, and developing methods for disease prevention. The use of nucleic acids, or aptamers, has emerged as more specific and accurate cancer diagnostic and therapeutic tools. Aptamers are single-stranded DNA or RNA molecules that recognize specific targets based on unique three-dimensional conformations. Despite the fact aptamer development has been mainly restricted to laboratory settings, the unique attributes of these molecules suggest their high potential for clinical advances in cancer detection. Aptamers can be selected for a wide range of targets, and also linked with an extensive variety of diagnostic agents, via physical or chemical conjugation, to improve previously-established detection methods or to be used as novel biosensors for cancer diagnosis. Consequently, herein we review the principal considerations and recent updates in cancer detection and imaging through aptamer-based molecules.

## 1. Introduction

Cancer is a major public health problem and the second leading cause of death worldwide. The number of new cases of cancer is expected to grow rapidly as the population increases and continues to make lifestyle choices that increase cancer risk [1,2]. According to Hussain and Nguyen, focusing on early detection and accurate staging of cancer is as important as the development of new anticancer drugs and treatments intended to cure cancer [3].

The accumulation of genetic alterations in cells together with abnormal cell division and elevated resistance to apoptosis are known factors in cancer development. These changes allow mutant cells to acquire the ability to overexpress certain proteins not expressed by normal cells [4]. These cancer-specific markers are attractive targets for cancer diagnosis and treatment [5]. Recently, greater emphasis has been placed on investigating not only improved therapeutics but also early detection and diagnosis methods. In many cases, cancer metastasizes throughout the body before it is diagnosed, resulting in decreased probability of successful cure and/or survival. Therefore, early and accurate cancer diagnosis is critical to increasing treatment efficacy, decreasing secondary diseases post-therapy, and ultimately developing methods for disease prevention [6,7]. Based on these observations, the necessity of more sensitive, low-cost, and/or non-invasive methods for cancer diagnosis is still a challenge for research laboratories.

Aptamers have recently emerged as potential molecular probes in the development of more specific and accurate cancer diagnostics and therapeutics due to their unique characteristics. Aptamers are single-stranded nucleic acid molecules (ssDNA or RNA) that recognize specific targets based on unique three-dimensional conformations [8]. A wide range of diagnostic agents can be linked to aptamers via physical or chemical conjugation in order to further customize their function [9]. Specifically, over the past decade, aptamers that target proteins overexpressed in tumors have become ideal tools for the specific recognition of these cancer-specific markers [10]. Consequently, the adaptation of aptamer-based technologies to actual cancer diagnosis methods could provide a great opportunity for advances in cancer detection. 

Thus, we present a state-of-the-art aptamer review with a specific focus on their application to cancer. We summarize the latest advances in aptamer use for cancer diagnosis, highlighting their potential future role as promising molecules in cancer detection and imaging.

## 2. Aptamers

**Figure 1:**
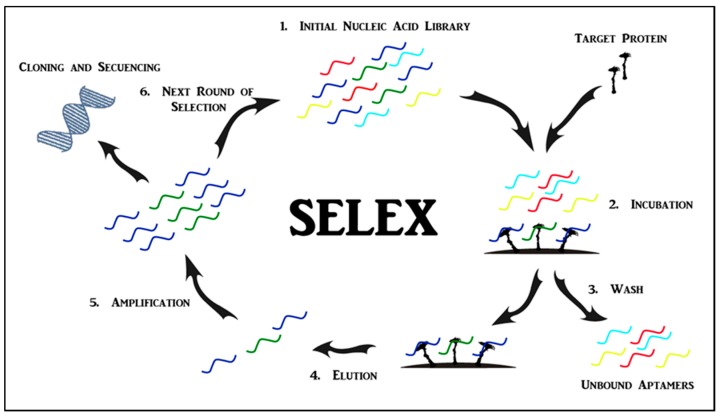
Systematic evolution of ligands by exponential enrichment (SELEX), for the generation of either single-stranded DNA or RNA oligonucleotides that bind to a target ligand. SELEX starts with the incubation of a nucleic acid library with the target protein. Unbound aptamers are washed off and protein-bound aptamers are eluted and amplified to create the library for the next round of selection. After iterative selection rounds, potential target-specific sequences are enriched and dominate the population of aptamers.

Aptamers are single-stranded nucleic acid molecules (ssDNA or RNA) that recognize specific targets based on unique three-dimensional conformations [8]. Aptamers are developed in vitro through a process called systematic evolution of ligands by exponential enrichment (SELEX) (Figure 1) [11]. SELEX technology was established by Tuerk et al. and Ellington et al. in 1990 [12,13]. Typically, a selection cycle starts with the incubation of the protein target with a combinatorial DNA or RNA library, comprised of approximately 10^12^–10^14^ unique oligonucleotide sequences 20–80 bases in length. Protein-bound aptamers are eluted and amplified to create the library for the next round of selection. An enriched pool of potential binders generated from PCR amplification is used in the subsequent selection rounds. To increase the selection stringency, a negative selection step can be introduced. After multiple selection rounds, potential target-specific aptamers are obtained [14]. Additionally, a negative selection step can be introduced to eliminate sequences that bind to non-desirable targets, thereby increasing the specificity of the selection [15,16].

The resulting highly enriched pool of aptamers can be analyzed using next-generation sequencing for further characterization of individual aptamers [17]. In fact, as better sequencing technologies and bioinformatic software are developed, aptamer researchers have been able to identify functional oligonucleotides from earlier rounds by evaluating population dynamics during selections [17,18,19]. Proteins are by far the most common target used in SELEX. However, the difficulty of generating high-purity recombinant human proteins, with native conformation as SELEX-targets, has made it difficult to produce more reliable and robust aptamers. In addition, SELEX is not applicable for unknown proteins, insoluble proteins, or proteins that only function in a multiprotein complex [20,21,22]. Consequently, scientists have been working hard on different variants of aptamer selection methodology. The advances and improvements made in traditional SELEX have allowed the development of new methods that permit the selection of aptamers against not only proteins [23] but also against a wide range of targets, such as live cells [24,25,26,27], viruses [28], bacteria [29], and tissues [30]. Cell-based SELEX methodology (cell-SELEX) stands out among other new aptamer-selection methods: the oligonucleotides selected with this technique are able to bind not just cell-surface molecules in their native state, but also unknown membrane receptors [31,32]. Therefore, cell-SELEX offers a new opportunity for working with aptamers that recognize the native state of proteins. 

Over the past two decades, cell-SELEX technology has been used for the selection of specific aptamers with diagnostic and therapeutic purposes over a wide variety of cell surface targets, particularly for tumor cells. Multiple groups have reported the discovery of new important biomarkers in different types of cancer and other diseases through the use of cell-SELEX [18,33,34,35].

The general protocol of cell-SELEX includes a positive selection step of incubation, elution, and amplification of binding aptamers, and a negative selection step is also necessary to remove sequences which bind to normal cells. In other words, the cell-SELEX method first starts with the incubation of the nucleic acid library with normal or control cells that express either normal surface proteins or do not express the target protein. This step is called the negative selection step. In the positive selection step, unbound aptamers are incubated with the target cancer cells. Candidate aptamers are recovered from the positive cell lines and amplified to become the library for the next round. This cycle of selection is repeated for as many rounds as necessary [36]. Changing the conditions of each selection round is crucial to increasing stringency and yielding high-affinity aptamers that bind to the target cell population [21,37]. The stringency can be increased after each cycle by increasing the washing steps and/or increasing the number of non-target competitors. Reducing the number of target cells will achieve the same goal, as it will force the aptamers to compete with each other for the target, increasing target affinity and the overall efficiency of the process. Finally, the enriched pools are sequenced, and representative aptamers are chosen for subsequent characterization. With the incorporation of flow cytometry and high-throughput sequencing to cell-SELEX technology, a shorter selection round and high-quality aptamer identification have been achieved [38,39].

Despite its enormous potential, cell-SELEX presents technical limitations such as cell conditions and the complexity of some cancer cell lines. It is important for the aptamer selection to avoid nonspecific uptake. Cell death leads to altered protein expression. Therefore, the elimination of these cells will favor the identification of specific aptamers. Second, it is important to choose the proper cell line because cell surface proteins are very complex in some cancers. Because of the complexity and heterogenicity of some cancer cells, it is critical to perform additional selection rounds against non-target cells in order to improve aptamer specificity. However, this selection process can be quite complex and may have a negative impact in terms of time and resources spent by the researcher [40].

Currently, new selection methods based on cell-SELEX have emerged to improve the success rate of aptamer selection, such as target expressed on cell surface-SELEX (TECS-SELEX) [41], fluorescence-activated cell sorting-SELEX (FACS-SELEX) [42], 3D SELEX [43], automated SELEX [44], click-SELEX [45], cell-internalization SELEX [46], and in vivo SELEX [24], among others.

Several factors need to be taken into consideration before the selection process begins. One of these aspects is the design of the library for SELEX procedures. Researchers must decide whether they are working with DNA or RNA aptamers—a decision that depends on the purpose of the selection. DNA and RNA aptamers are functionally similar, but each has its own unique benefits. DNA aptamers are inherently more stable, cheaper, and easy to produce. RNA aptamers typically have more diverse three-dimensional conformations due to the 2′-OH group and stronger RNA-RNA interactions, which probably increase binding affinity and specificity. However, RNA aptamers need more steps between selection rounds than DNA aptamers, making the method potentially more cost- and labor-intensive. Whereas PCR is used to recover the DNA aptamer-bound sequences, RNA aptamers must first be reverse-transcribed into cDNA to enable subsequent PCR amplification and RNA in vitro transcription for the next selection round [15,47].

There are also several challenges in terms of in vivo application that remain unresolved. Aptamers are vulnerable to nuclease degradation and have short circulation times. They can be chemically modified to enhance their nuclease resistance and in vivo stability. Replacing the 2′-OH position with fluoro (F), amino (NH_2_), or *O*-methyl (OCH_3_) groups can increase the half-lives by reducing aptamer degradation. Capping the 3′end with an inverted dT residue can provide resistance to serum nucleases as well. The latest results of Kratschmer and Levy indicate that fully modified oligonucleotides (100% 2′-*O*-Methyl or 2′-*O*-Methyl A, C, and U in combination with 2′-fluoro G) had the longest half-lives. They also demonstrated little degradation in human serum even after prolonged incubation [48,49,50]. In practice, these modifications can be accomplished by two different methods: in-SELEX and post-SELEX. In the in-SELEX strategy for aptamer selection, a DNA or RNA library containing the modified nucleotides is produced by using a mutant T7 RNA polymerase [51,52]. However, according to Rozemblum et al., backbone modifications can reduce the ability of polymerase to amplify the DNA in the PCR amplification step [53]. Conversely, the post-SELEX strategies introduce modifications to aptamers after the selection, during the solid-phase chemical synthesis of individual aptamers [54]. To solve the problem of renal filtration, aptamers are generally linked to polyethylene glycol (PEG), cholesterol, or proteins. Using a multivalent aptamer design can also help to achieve the same goal [55,56,57]. Regarding toxicity, while it is very limited in humans, chemical modifications should be used carefully in order to avoid undesired effects. In the Phase III study of the Regado Biosciences aptamer-based anticoagulation system (REG1 system) [58], serious allergic responses to PEG were observed [59]. Thus, evaluation and optimization of aptamer-modifications are still a challenge.

Several authors have reviewed the importance of aptamer development and research by emphasizing their advantages over antibodies [60,61,62]. However, the clinical success of aptamers is far behind antibodies, with only one FDA-approved aptamer available to-date [63]. The use of antibodies in cancer diagnosis and therapy has dominated the marketplace, while aptamer development has remained mainly confined to laboratory settings. Nevertheless, aptamers are still notable molecules with great potential for clinical application. Instead of comparing them with antibodies, scientists should rather concentrate on the unique attributes of aptamers and take advantage of them. Aptamers present excellent stability to variations in pH, temperature, and ionic conditions of the environment [64]. They have low immunogenicity and toxicity, and they are easily synthesized and modified in large quantities without batch-to-batch variation [64,65]. Furthermore, the size of the aptamer enables deeper tumor penetration [66]. They can be selected over a wide range of targets: ions, organic and inorganic molecules, nucleic acids, proteins, peptides, toxins, viral particles, whole cells, entire organs, and live animals [64]. More importantly, aptamers could be used as a delivery tool to release diagnostic or therapeutic cargoes into the intracellular space of target cells. In this way, aptamers could be linked with drugs, non-coding RNAs, proteins, and nanoparticles, making them promising tools for specific tumor diagnosis and therapy [67].

## 3. Applications of Aptamers in Cancer Imaging and Diagnostics

For the past decade, researchers have been working in the development of new methods for cancer imaging and diagnosis that can identify the presence of cancer biomarkers at early stages, before the appearance of any symptoms. Being able to detect cancer early on would allow for earlier treatment responses and increased probability of curing the disease. Nowadays, physicians use magnetic resonance imaging (MRI), computed tomography, and other imaging techniques combined with flow cytometry, immunohistochemistry, and cancer biomarker assays in serum to reach a diagnosis in cancer patients [68]. However, these methods face various technical issues, especially due to the often low concentrations of cancer markers mixed with other proteins and cells that make their identification in bodily fluids quite difficult. These methods can also be time- and labor-intensive [69,70,71]. Consequently, aptamers appear as a promising tool for cancer imaging and detection in biological samples because they are able to recognize almost any oncological biomarker, cancer metabolite, or cancer cell with high affinity and specificity at low target concentrations. Researchers may also benefit from the adaptability and easy chemical manipulation of aptamers. Thus, aptamers could be linked with a wide range of existing cancer detection and imaging platforms to improve sensitivity and selectivity [6,72]. They could also be used to detect and measure the expression or activity of target molecules that influence tumor behavior or change in response to therapy [73].

### 3.1. Aptamer-Nanoparticle System

**Figure 2:**
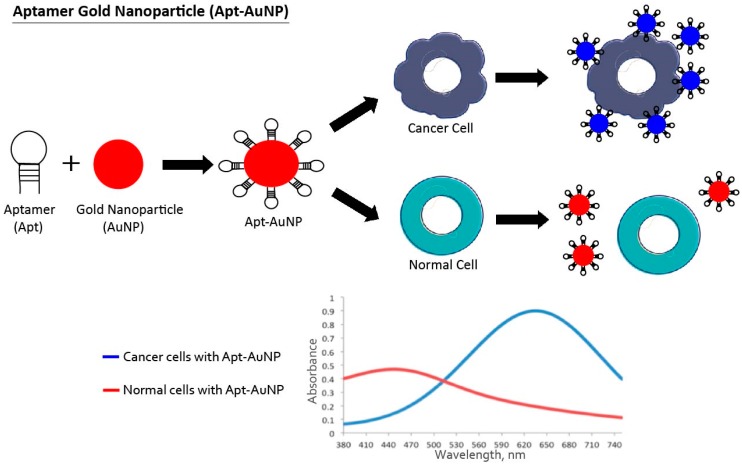
Schematic illustration of aptamer-gold nanoparticle (Apt-AuNP) molecules for cancer cell detection. The aptamer (Apt) linked to gold nanoparticles (AuNP) bind specifically to cancer cells and start to behave as a larger gold structure because of the particle’s proximity. The short proximity to each other causes a shift in the absorption spectra of the particles. However, the Apt-AuNP complex cannot recognize normal cells, which results in no alteration of the absorption signal.

**Figure 3:**
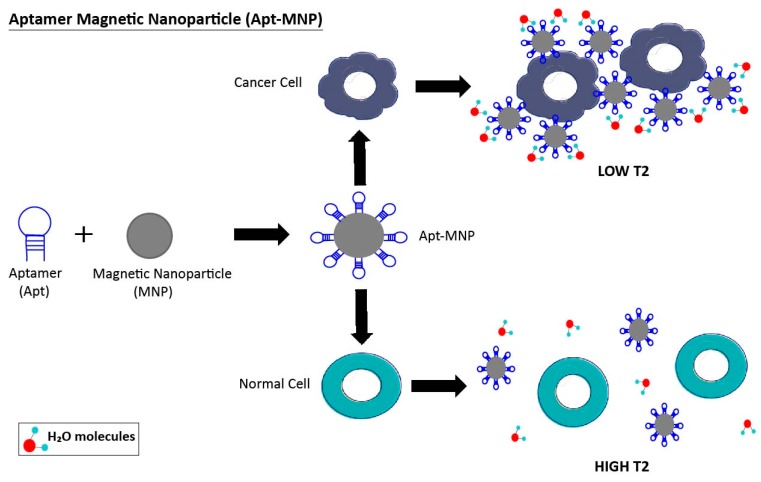
Aptamer-magnetic nanoparticle (Apt-MNP) clusters bind to cancer cells and lead to the aggregation of these magnetic molecules. The accumulation of Apt-MNPs around tumor cells cause a decrease in the T2 of adjacent water protons. Whereas, in the presence of normal cells, Apt-MNPs are well dispersed, resulting in a high T2 of surrounding water protons. T2 = spin-spin relaxation time or transverse relaxation time is a measure of the rate of decay of transverse magnetization within the x-y plane, and is reported in milliseconds.

One detection system used for cancer diagnosis is aptamers linked to nanoparticles (Apt-NP). The use of this chimera is a good alternative to achieve the specific extraction and detection of cancer cells in complex fluids such as blood. Due to the low molecular weight of the aptamer, this method allows cell-specific aptamers to be immobilized on the surface of nanoparticles to create an aptamer–gold nanoparticle (Apt-AuNP) system (Figure 2) or aptamer-magnetic nanoparticle (Apt-MNP) cluster (Figure 3), among others. These Apt-NP chimeras are expected to improve cancer diagnosis with higher sensitivity and selectivity compared to cancer-cell-detection strategies that use aptamers or nanoparticles alone. Interestingly, nanomaterials protect aptamers from being digested by nucleases [29,74].

Liu et al. developed an aptamer-conjugated gold nanoparticle strip biosensor (ANSB) for the detection of Ramos cells, even in human blood [75]. In short, Liu et al. used a pair of previously identified aptamers through cell-SELEX for Burkitt’s lymphoma [33], a thiolated aptamer TD05 (Thiol-TD05), and a biotinylated aptamer TE02 (Biotin-TE02). In this work, both aptamers Thiol-TD05 and Biotin-TE02 were immobilized on the nanoparticle surface and on the test zone of ANSB, respectively.

A sample containing Ramos cells and Thiol-TD05-NP migrate along the strip. The Thiol-TD05-AuNP-Ramos cell complex is captured on the test zone through the immobilized aptamer, and its accumulation produces a quantifiable red band. As a result of ANSB, approximately 4000 Ramos cells could be detected with the researcher’s eye and 800 cells with a portable strip reader. In similar studies, Medley et al. and Borghei et al. developed a colorimetric assay based on the color produced by the conjugated aptamer-nanoparticle [76,77]. In Medley’s study, a thiol-aptamer for T- acute lymphoblastic leukemia cells was linked to gold-nanoparticles. When the Thiol-Apt-AuNP recognized the target cells, a change in color was observed. Thus, by measuring the color change, researchers were able to differentiate between target and non-target cells. Borghei et al. used the ability of the target cells to capture the aptamer. A specific aptamer for nucleolin, a protein overexpressed in metastatic cells, was used. When the sample contained the cancer cells, the aptamer bound nucleolin receptors. The solution containing the single-stranded DNA (ssDNA) joined to gold nanoparticles, revealing a red color because of the aptamers’ removal. However, when aptamers, but no cancer cells, were present in the sample, the solution was blue because aptamers interact with ssDNA-AuNP probes. This procedure had a detection limit of 10 MCF-7 cells. In contrast, Hu et al. developed an approach in which the aptamer and the nanoparticle were separated but interacted with each other via biotin-streptavidin interaction [78]. In this study, HepG2 cells were incubated with biotin-conjugated TLS11a aptamer (Bio-TLS11a). Then, the cancer cells-Bio-TLS11a complexes with streptavidin-labeled fluorescent silica nanoparticles (SA-FSNPs) were detected due to strong and specific biotin-streptavidin association. This approach allowed the sensitive and specific detection of hepatoma cancer cells in vitro. Moreover, an aptamer-modified fluorescent silica nanoparticle (FSNPs) system was used by Tan et al. for the detection of leukemia cells. This group used Sgc8 aptamer labeled with amine linked to carboxyl-modified FSNPs. This system could detect leukemia cells with high sensitivity and specificity [79]. Finally, the same Sgc8 aptamer, as well as 41t and TE17 aptamers, were linked to a sophisticated nanorobot and tested in response to target proteins. When aptamers recognized their targets, active previously-loaded cargoes such as gold nanoparticles, were delivered to the target cell. This study demonstrated the promising future of aptamers for the specific delivery of nanoparticles loaded into DNA-based nanorobots for cancer cell detection [80].

A similar approach to an Apt-AuNP system was pursued using aptamers conjugated to magnetic nanoparticles (Apt-MNPs). Tan’s group developed an effective method for the detection of a minimum of 10 cancer cells in 250 μL of sample. The method consists of measuring the change of spin-spin relaxation time (ΔT2) of the surrounding media water protons. It is well-known that MNPs alone enhance the magnetic resonance signal of protons from surrounding water molecules. In this way, when aptamer-magnetic nanoparticle clusters recognize the cancer cells, they generate a magnetic field that brings a consequent decrease of the T2. Tan et al. were able to recognize human T-leukemic cells (CCRF-CEM) and colorectal cancer cell lines (DLD-1 and HCT 116) using specific aptamers for each cell line in different complex biological media, including fetal bovine serum, human plasma, and whole blood. This proof-of-concept study demonstrated the ability of the Apt-MNPs clusters to be a sensitive and specific method for cancer cells detection and diagnosis [81].

A different approach was developed combining the biological recognition property of Apt-AuNP with a physical transduction technique (i.e., electrochemiluminescence, ECL), to achieve the detection of target cancer cells [82,83]. Zhang et al. could detect cancer cells using an Apt-magnetic complex and a reporter DNA-AuNP combined with a sensitive ECL approach. In this study, a DNA-1 was hybridized in a CdS nanocrystal (NC) film. HL-60-specific aptamers were linked to magnetic beads (Apt-MBs) and hybridized with a DNA-2 labeled with AuNPs. In the presence of HL-60 cells, the Apt-MBs conjugated with the cell surface and the DNA-2-AuNPswere released. After magnetic separation, the DNA-2-AuNPs hybridized with the captured DNA-1 on the CdS NC film, increasing the ECL signal. Between 20 to 1.0 × 10^6^ cells/mL of HL-60 cells could be detected using this approach. In another study, Zhang et al. demonstrated the electrochemical detection of CCRF-CEM cells using Apt-MNPs conjugated with ferrous ferric oxide (Fe_3_O_4_). A competitive binding assay was performed in which T-acute leukemia cells competed efficiently with an AuNP-conjugated cDNA for binding specifically to the aptamer of the Fe_3_O_4_-Apt-MNP complex. The released AuNPs were treated via silver deposition in the presence of target cells. The AuNP-catalyzed silver deposition enhancement showed high sensitivity with a detection limit of 10 cells [84]. Another highly sensitive and selective method for the early detection of leukemia cells was developed by Khoshfetrat and Mehrgardi [85]. In this study, the T-cell leukemia sgc8c aptamer, a truncated version of sgc8 aptamer, was linked to gold nanoparticles-coated magnetic Fe_3_O_4_ nanoparticles (Apt-GMNPs). Ethidium bromide (EB) was intercalated into the stem of the aptamer hairpin. The presence of cancer cells caused the disruption of the hairpin structure of the aptamer, forcing the release of EB and a consequent decrease in electrochemical signal. Under optimal conditions, this technology could detect a range of leukemia cancer cells from 10 to 1 × 10^6^ cell/mL. However, most of the ECL approaches achieve cell detection by capturing cells on the electrode surface. A novel sandwich sensing system through an aptamer-cell-aptamer architecture has been developed by several authors [86,87,88,89]. In their studies, one of the aptamers was combined with AuNPs and immobilized on the electrode surface. Cancer cell lines could bind to this sandwich sensing system, and they could be measured by cyclic voltammetry (CV) and electrochemical impedance spectroscopy (EIS) using ferricyanide (Fe(CN)_6_^3^^−/4^^−^) as a redox probe. More recently, Crulhas et al. described a system to detect vascular endothelial growth factor (VEGF) and prostate-specific antigen (PSA) released by prostate cancer cells based on specific thiolated aptamers bound to a gold-covered electrode surface [90]. This electrochemical aptamer-based biosensor could detect 0.08 ng/mL of PSA and 0.15 ng/mL of VEGF, released in vitro by three different prostate cancer cell lines (RWPE-1, LNCaP, and PC3). The precise detection of these biomarkers make this aptasensor a potentially important non-invasive tool for prostate cancer diagnosis. It is clear that the use of aptamer-based electrochemical biosensors is gaining popularity due to its simplicity, rapid response, low-cost fabrication, and high sensitivity [91].

### 3.2. Aptamer-Microfluidic Devices

**Figure 4:**
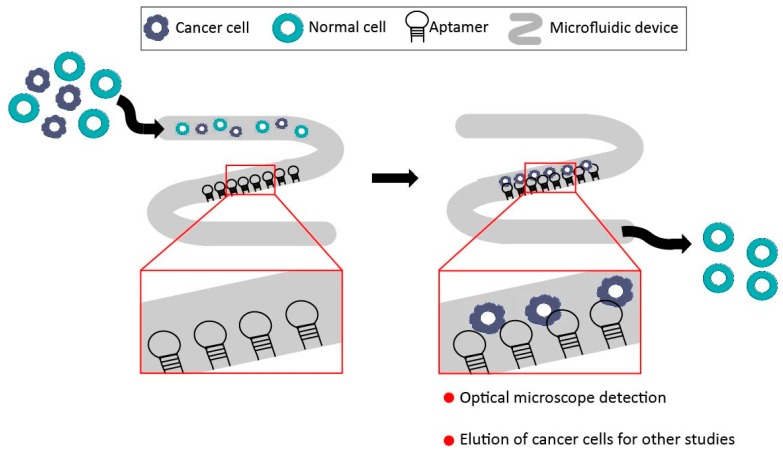
Schematic illustration of a microfluidic device, showing a middle region with immobilized aptamers. After the injection of the sample, cancer cells are trapped by specific aptamer-cell interaction, whereas normal cells pass through the device. In this sense, cancer cells can be detected by optical microscope observation of the middle zone, and also eluted for future experiments. This device allows the isolation of cancer cells from a heterogeneous mixture of cells by selective cell-capture of immobilized aptamers.

The use of aptamers for cancer diagnosis has also spread to other platforms. In 2009, Phillips and colleagues revealed the combination of aptamers with microfluidic devices to be an excellent platform for cancer diagnosis, since this device is cheap, simple, and able to detect multiple cancer cells [92]. Briefly, aptamers are first immobilized on the surface of a microfluidic channel. Then, a mixture of cells is passed along this device, allowing specific immobilized aptamers to capture target cells (Figure 4). The percentage of captured cells can be measured by optical microscopy. Xu et al. proved that captured cells could be sorted and cultured for further studies using this platform [93]. To achieve this goal, three different aptamers for leukemia cells (Sgc8, TD05, and Sgd5) were immobilized on the surface of the microfluidic device and the leukemia cells were isolated with high purity (50% to 100% purity for the different leukemia cell lines). In a similar study, a DNA aptamer against protein tyrosine kinase 7 (PTK7), a protein overexpressed in multiple human cancers, was reportedly immobilized on a microfluidic surface [94]. An aptamer-3D network was synthesized in this study using rolling circle amplification (RCA) to increase the capture and isolation of the target cells. RCA is a DNA amplification technique in which short DNA is elongated with the assistance of a circular DNA template. Consequently, RCA is employed to extend the aptamer sequence, creating a 3D network with multiple aptamer domains on the microfluidic surface. CCRF-CEM cells (T-human acute lymphoblastic leukemia cells) were captured with higher efficiency than monovalent aptamers and antibodies immobilized with the same device. Captured cells could be easily released for further analysis using restriction enzymes to cleave the aptamers. Interestingly, other authors have also incorporated the specific cell aptamer recognition with the RCA technology in their experiments to augment the specificity and sensitivity for the detection of cancer cells [95,96]. Recently, Chen et al. investigated how cells and aptamers interact under different flow conditions in microfluidic devices [97]. They used T- acute leukemia cells (CCRF-CEM) and their specific aptamer, Sgc8, to analyze the effects of flow rates and device shapes. The results of CCRF-CEM capture efficiency in this study will help researchers to choose the best experimental conditions and device shapes. Another elegant study conducted by Sheng et al. used DNA nanostructures combined with microfluidics for circulating tumor cell (CTCs) isolation [98]. In this work, DNA nanospheres were constructed by immobilizing up to 95 aptamers to gold nanoparticles (AuNP), and then these DNA nanospheres were immobilized onto the channel of microfluidic devices. Efficient isolation of CTCs from cell mixture and whole blood was achieved using this platform. This study showed that the combination of two different technologies, nanotechnology and microfluidics, has enormous potential in the cancer field.

### 3.3. Aptamer-Quantum Dots Probes

**Figure 5:**
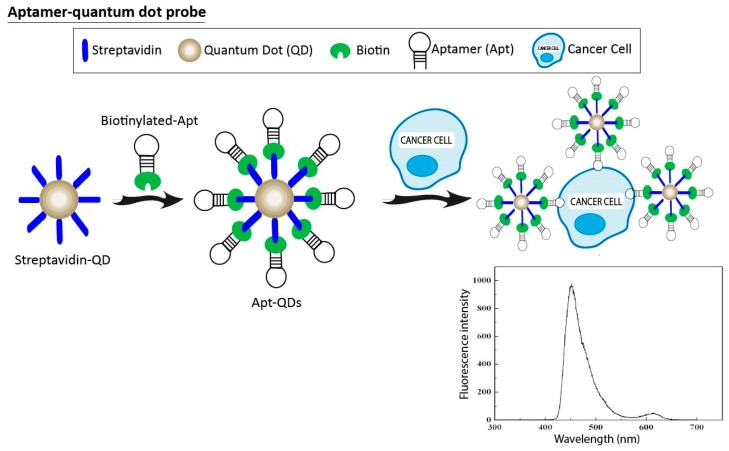
Schematic representation of functionalized aptamer-quantum dot (Apt-QD) probe. A group of biotinylated aptamers (biotinylated-Apt) that target a cancer cell are conjugated with streptavidin-quantum dot (Streptavidin-QD) via biotin-streptavidin-specific interaction. When Apt-QDs recognize the cancer cell, the fluorescence emission spectra of QDs is observed.

Quantum dots (QDs) appear to be promising tools for cancer imaging and diagnosis because they can be easily conjugated with nucleic acids that specifically target biomolecules. QDs with distinct emission wavelengths can be ligated to different aptamers for multiple cancer detection (Figure 5). Kang et al. presented a system that simultaneously targets three cancer molecular markers: tenascin-C, nucleolin, and mucin (MUC-1). To achieve this goal, three aptamers (TTA1, AS1411. and MUC-1) were conjugated to QDs with distinct emission wavelengths of 605, 655, and 705 nm, respectively. Healthy and cancer cell lines were incubated with QD-aptamer conjugates. Results showed that these QDs-aptamer complexes could not only produce a visible fluorescence signal in the presence of target cells, but could also differentiate between different types of cancer cells [99]. Similarly, Lian et al. used the AS1411 aptamer conjugated with QDs to recognize breast cancer cells through confocal microscopy, being suitable for in vitro diagnostic biosensing [100]. In 2015, two different aptamer-conjugated QDs (TTA-1 and AS1411 aptamer) were used by Lee et al. to simultaneously demonstrate the presence of the cancer biomarkers tenascin-C and nucleolin in different cancer cell lines [101]. In similar studies, two different researchers created polymeric structures by conjugating acrylamide, aptamers, and QDs [102,103]. These polymeric QD-aptamer systems successfully exhibited fluorescence in the presence of cancer cells. Furthermore, they proved to be suitable systems for long-term fluorescent cellular imaging. Recently, Tang’s group developed a new system for use in fluorescence-guided surgery for glioma, using A32 aptamer labeled with QDs. A32 aptamers specifically recognized epidermal growth factor receptor variant III, highly dispersed on the surface of glioma cells. The system generated strong fluorescence in the mouse model of glioma, and was nontoxic both in vitro and in vivo. Most importantly, this system could be applied for preoperative diagnosis and postoperative examination of glioma [104]. Generally, aptamers are first labeled to QDs. Then, the aptamer-QDs recognize the cancer cells. However, Wu et al. developed a recognition-before-labeling strategy, where the aptamer first recognizes the target cells and then fluorescent QDs bind to the aptamers-cell conjugate via simple streptavidin-biotin interaction. This new method for in vitro diagnostic assays of cancer cells has some advantages over other strategies because it avoids the impact on aptamer configuration when conjugated to QDs [105].

### 3.4. Molecular Beacon Linked to Aptamers

**Figure 6:**
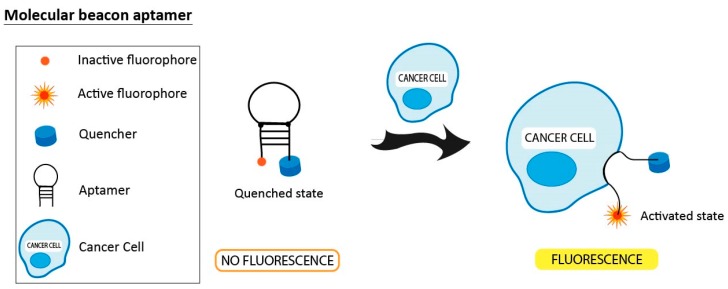
Molecular beacon aptamers (MB-Apt) for cancer cell detection with fluorophore and quencher at the 5′- and 3′-ends of the aptamer, respectively. In the absence of a target, the hairpin structure of the MB-Apt holds the quencher molecule close to the fluorophore, resulting in no fluorescence emission. When the MB-Apt is bound to membrane receptors of the cancer cell, its conformation is altered, thus resulting in an activated fluorescence signal because of fluorophore-quencher physical separation.

Molecular beacon aptamers have been used to monitor different kinds of targets, including cancer cells. A molecular beacon (MB) is a dual-labeled single stranded DNA with a fluorophore at the 5’ end and a quencher at the 3’ end. The molecule has a stem-loop structure where the fluorophore and quencher do not fluoresce when in close proximity to each other. However, if the probes hybridize to a target sequence, separating the fluorophore and quencher, they will fluoresce (Figure 6). Shi et al. developed an activated fluorescence aptamer probe using molecular beacon technology [106]. While fluorescence is quenched in free Sgc8 aptamers, fluorescence is activated after targeting CCRF-CEM cancer cells due to an aptamer-conformational alteration. Activated fluorescence signals were observed in vitro and in vivo in the CCRF-CEM tumor sites. Another proof-of-concept study conducted by Zeng et al. [107] featured an aptamer-reporter conjugated with a pair of fluorochrome-quencher molecules that selectively target circulating tumor cells (CTCs). In the absence of CTCs, the probe is optically silent. However, when it recognizes and is taken up by tumor cells, the aptamer-reporter is rapidly degraded, resulting in the separation of the fluorochrome and quencher, with the subsequent activation of the fluorochrome. This method was used to detect CTCs with no background noise in whole blood and bone marrow aspirates. Using the same principle, aptamers were used by different researchers as internalizing carriers for the specific delivery of MB probes targeting mRNA and miRNAs. So far, the AS1411 aptamer was used labeled with a MB probe specific for miRNAs. Both Li et al. and Kim et al. used the great cancer selectivity of AS1411 to achieve cell-specific delivery of the MB probe that allows intracellular miRNA imaging of the miRNA target [108,109]. Interestingly, aptamer-MB for miRNA imaging could be applied to other cancers by changing the target miRNA sequence of the MB. Another study conducted by Qui and colleagues investigated intracellular mRNA analysis in live cells using aptamer-based molecular beacon probes. The internalizing aptamer AS1411 with an extended cDNA sequence was linked to a single-stranded MB specific for the mRNA. As expected, the MB was delivered into the cytoplasm of cancer cells, allowing detection of the mRNA after fluorescence was activated [110].

Two years ago, Zhang et al. identified abnormal DNA methyltransferase activity which is closely associated with cancer, through the detection of DNA adenine methylation methyltransferase (Dam MTase) activity [111]. Both a streptavidin-specific aptamer (SA-apt) labeled with a single fluorophore and an allosteric molecular beacon (aMB) were used in this study. The SA-apt could not bind with SA beads because a stable hairpin structure was formed in the absence of a target. Nevertheless, the presence of Dam MTase methylated the aMB, making it available for the restriction nuclease Dpnl to cut the methylated probe and release the fluorophore-labeled aptamer. The free SA-apt bound to SA beads, making the beads highly fluorescent. DNA methyltransferase activity can be quantified by a microscope or by flow cytometry. Zhao’s group similarly took advantage of conformational changes after binding to develop an “activatable” aptamer-based fluorescence probe (AAFP) to detect cancer cells and frozen cancer tissue [112]. In this study, the TLS11a aptamer, specific for HepG2 cells, was linked to two short-complementary DNA sequences, with a 5’-fluorophore and 3’-quencher, respectively. In the absence of a target, the AAFP formed a hairpin structure capable of auto-quenching. In the presence of cancer cells, the fluorophore and quencher separated, making the AAFP emit a strong fluorescence signal. Under optimal incubation conditions, AAFP was able to detect cancer cells at concentrations as low as ~100 cells/mL. These results indicate the AAFP could be a promising tool for the specific detection of cancer cells with low signal-to-background ratio. Another elegant study designed by Hwang et al. detected the exogenous EpCAM (epithelial cell adhesion molecule) or muc1 (mucin1) expression correlated to cancer metastasis [113]. In this study, a quantum dot-based aptamer beacon was used for CTC diagnosis, conjugated with a 5’-quantum dot and a 3’-black hole quencher. In the absence of target EpCAM/muc1 of CTCs, the aptamer remained in the quenched state because of its conformational state. When the target molecule was present, a fluorescence signal was emitted due to the activation of the EpCAM/muc1 aptamer. A change in its conformation, when targeting EpCAM/muc1, allowed separation of the QD and the quencher and the subsequent fluorescence signal emission.

### 3.5. Fluorescent Aptamer Probes

**Figure 7:**
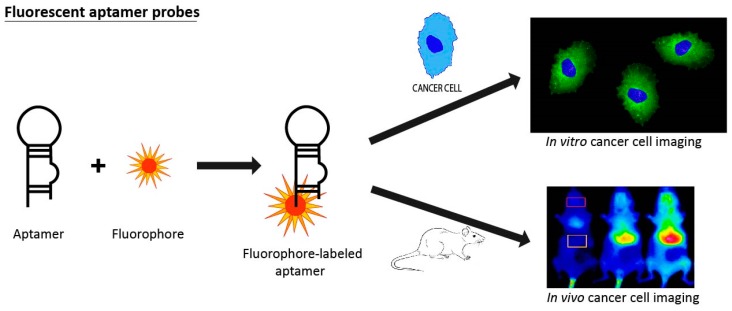
Illustration of fluorescent aptamer probes. The aptamer is labeled with fluorochrome at its 5′ end. This chimeric molecule could be used for fluorescence staining of cancer cell as the top image, as well as in vivo cancer cell imaging in tumor-bearing mice, as in the picture beneath.

Fluorescent aptamer probes appear to be one of the most widely used imaging tool in aptamer research due to their low cost and high sensitivity (Figure 7). One of the first authors to apply fluorescent aptamer probes to cancer research was Zhang et al., who used a fluorescent-labeled RNA aptamer specific for CD30—a protein overexpressed in lymphoma cell lines [114]. Specific binding of the aptamer to lymphoma cells was confirmed using flow cytometry and fluorescence microscopy. While the anti-CD30 antibody is currently the gold standard for CD30 detection, this study suggests that CD30 aptamer could be used in combination with CD30 antibody for the improved detection and diagnosis of lymphoma. A study by Shi et al. used a novel aptamer-based fluorescence imaging approach to selectively detect Ramos tumor in mice using Cy5-labeled TD05 aptamer (Cy5-TD05), specific to Ramos cells (B-cell lymphoma cell line). Cy5-TD05 was injected intravenously into Ramos tumor-bearing nude mice. The fluorescent probe could effectively recognize Ramos tumors and determine their spatial and temporal distribution, up to 5–6 h after binding the targets. This study was the first to use aptamers obtained through cell-SELEX for in vivo fluorescence imaging [115]. Later, the same group developed an aptamer-based fluorescence probe for human lung cancer imaging using Cy5-labeled S6 aptamer obtained using whole cell-SELEX [116]. These aptamers specifically targeted A549 lung carcinoma cells in both buffer and serum. After Cy5-S6 was intravenously injected into nude mice, the aptamer recognized, with high specificity, A549 lung carcinomas over Tca8113 tongue carcinomas (off-target), presenting a clear imaging result for in vivo fluorescence molecular imaging of carcinomas. In the same study, two aptamers for liver carcinoma cells recognition, LS2 and ZY8, were used to confirm the efficacy of the whole-cell SELEX method in generating molecular imaging probes that target different cancer types and even subtypes in complex systems. Another good example of fluorescent-labeled aptamers is the J3 aptamer for metastatic cancer. Yuan and colleagues identified a new DNA aptamer named J3, specific for the metastatic colorectal carcinoma LoVo cells through cell-SELEX [117]. The Cy5-labeled J3 aptamer was able to recognize colorectal carcinoma metastasis with a detection rate of 73.9%, whereas a small percentage of non-metastatic colorectal carcinoma cells were recognized using the J3-Cy5 aptamer. These results illustrate the exciting potential of J3-Cy5 for clinical diagnosis of cancer metastasis. Another potential target for fluorescent-labeled aptamers, human matrix metalloprotease-9 (hMMP-9), recently emerged due to its overexpression in malignant tumor cells, especially in cutaneous malignant melanoma. Kryza et al. evaluated the chemically modified RNA aptamer F3B as an imaging agent for malignant tumor diagnosis [118]. In this study, both fluorescent- and isotope-labelled aptamers were used to evaluate, both ex vivo and in vivo, the target efficiency of F3B in melanoma diagnosis. Optical fluorescence imaging and isotope tumor uptake confirmed the specific binding to hMMp-9 protein in A375 melanoma-bearing mice. The specificity of F3B was also confirmed ex vivo in human melanoma samples. The results of this study, together with the previous studies, indicate that fluorescent-labeled aptamers have excellent potential to improve upon current tumor imaging methods.

### 3.6. Aptamers in MRI Technology

**Figure 8:**
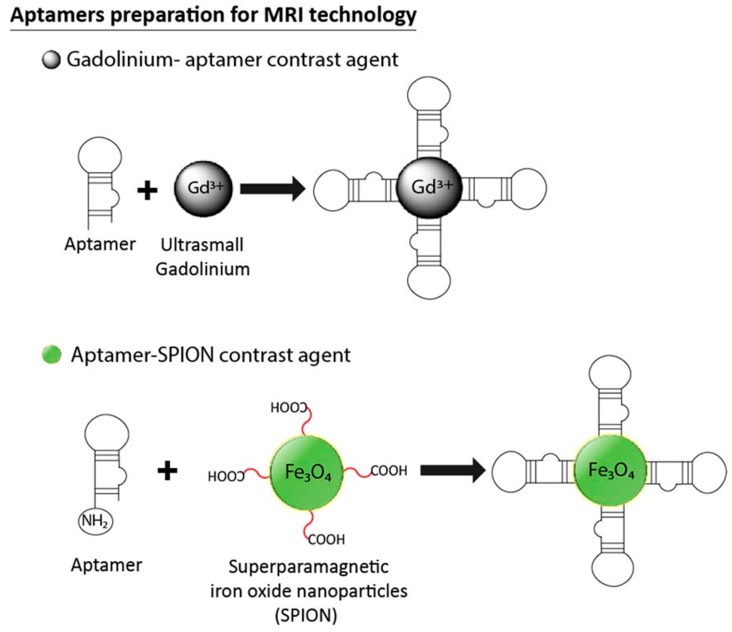
Schematic depiction of aptamer-gadolinium and aptamer-SPION nanoprobes for cancer cell detection with magnetic resonance imaging (MRI) technology. The aptamer can conjugate with gadolinium (Gd(III)) or magnetite (Fe_3_O_4_) nanoparticles via strong and specific interactions between the aptamer and the nanoparticle. In this sense, the chimeric molecule could be used as a contrast agent because it could change the magnetic relaxation time in a measurable way when targeting the cancer cell.

Magnetic resonance imaging (MRI) is an imaging technique that uses the behavior of protons in a magnetic field to construct 3D images of biological systems. In order to improve the sensitivity of this technique, small exogenous probes based on gadolinium (Gd(III)) or manganese (Mn(II)) complexes could be used as contrast agents due to their measurable influence on magnetic relaxation time (T1 and T2) [119]. Over the last decade, researchers have explored the use of aptamers with MRI. These smart vectors could be linked to contrast agents for target-specific molecular and cellular imaging (Figure 8). Li et al. were able to specifically identify MCF-7 cells by coupling MRI and fluorescence imaging in vitro. Fluorescence reporters made with AS1411 aptamer silver nanoclusters (aptamer-Ag NCs) were conjugated with PEG-Gd_2_O_3_ nanoparticles (NPs) and used as MRI contrast agents. The formation of PEG-Gd_2_O_3_/aptamer-Ag NCs nanoprobes demonstrated their application as multimodal molecular imaging probes by enhancing the fluorescence emissions of each molecule in vitro [120]. Newer contrast agents such as superparamagnetic iron oxide nanoparticles (SPIONs) appear to be more promising than traditional gadolinium-based MRI contrast agents due to their lower toxicity and detection limits. In 2008, Wang et al. linked the A10 RNA aptamer with SPION for prostate cancer cell imaging in vitro [121]. They showed a dramatic decrease in the longitudinal and transverse relaxation times (T1 and T2) when interacting with prostate membrane antigen (PSMA)-positive cells, while only a small change in T1 and T2 was observed in control cells. Additionally, the chemotherapeutic agent doxorubicin (DOX) was intercalated with the aptamer-SPION complex for PSMA cell therapy. The SPION-Apt was found useful for the detection and treatment of prostate cancer cells in vitro. Three years later, the same RNA aptamer-SPION-DOX construct was evaluated both in vitro and in vivo for MRI detection and therapy [122]. This complex was found effective for the detection of prostate cancer cells using MRI. It was also able to deliver DOX to the tumor site and monitoring the response of tumors in a mouse model. Another elegant study used the same contrast agent, ultrasmall superparamagnetic iron oxide nanoparticles (USPION), linked to the tumor vascular endothelial growth factor 165 aptamer (VEGF165-aptamer), for in vitro and in vivo MRI imaging [123]. In this study, the binding activity of the probe was assessed in vitro, then in vivo with liver cancer cells that express VEGF165 in a mouse model. The results indicated that the imaging effect could be seen within 3 h after the administration of the probe, but disappeared after 6 h, making their application in vivo quite promising. In another study, Fe_3_O_4_ nanoparticles with a fluorescent silicon dioxide (SiO_2_) shell (MFS) were conjugated to TLS11a aptamers for liver cancer-cell-specific targeting and imaging [124]. The use of HepG2 cells demonstrated the specific uptake of the aptamer-conjugated nanoprobe by both fluorescence and MRI. These results were confirmed in tumor-bearing mice in vivo with MRI images of the liver at various time points. The nanoprobe exhibited low toxicity and good biocompatibility, making it a good candidate for further studies in the biomedical imaging field. A similar study conducted by Keshtkar et al. used a construct for the detection of nucleolin-expressing breast cancer cells [125]. The MRI results showed a statistically significant difference in the signal intensity of the aptamer-conjugated Fe_3_O_4_-Au nanoparticle when interacted with different cancer cell lines. The authors concluded that the designed nanoprobe could both specifically bind breast cancer cells (4T1 cells) and be used as an MRI contrast agent.

## 4. Aptamers in Clinical Trials

Despite the vast number of aptamers mentioned in this review (Table 1), only a few have reached clinical trials for cancer diagnosis. The Sgc8 aptamer is currently being evaluated as a specific imaging agent in healthy volunteers and colorectal cancer patients [126]. The study began in 2017 and is expected to conclude in 2019. Additionally, Landman’s group conducted an observational clinical trial for bladder cancer detection [127]. This investigation aims to develop novel molecular sensors specific for urinary biomarkers of bladder cancer. Furthermore, in 2017, Aptamer Sciences (Gyeonggi-do, Republic of Korea) started commercializing a panel to detect non-small cell lung cancer using an aptamer-based protein biomarker technology [128]. Jung et al. conducted the study, and a total of 200 clinical samples were assessed to develop and validate the test, showing a 75% sensitivity and a 91% specificity with benign nodules controls.

Regarding clinical trials for oncology treatment, several aptamers have undergone clinical evaluation for the treatment of different diseases. However, only one aptamer-based drug was approved by the Food and Drug Administration (FDA). In 2004, the anti-vascular endothelial growth factor (VEGF) aptamer, pegaptanib (Macugen^®^) received FDA approval for age-related macular degeneration (AMD) therapy [63]. This RNA aptamer was soon relegated by more effective antibodies for AMD treatment, such us bevacizumab (Avastin^®^) [129], ranibizumab (Lucentis^®^) [130], and aflibercept (Eylea^®^) [131]. 

Two aptamers for cancer therapy have failed to pass clinical trials: AS1411 [132] and NOX-A12 [133]. The first aptamer to undergo a clinical trial for cancer therapy was AS1411, a nucleolin-specific DNA aptamer. Its evaluation in Phase II clinical trials showed a minimal response to treatment in both acute myeloid leukemia and renal carcinoma patients [134,135]. The spiegelmer NOX-A12 aptamer, an L-form RNA aptamer specific against C-X-C chemokine ligand 12 (CXCL12), has also been implicated in two different clinical trials. In a Phase II clinical trial for the treatment of chronic lymphocytic leukemia (CLL), NOX-A12 was evaluated in combination with bendamustine and rituximab [136]. Additionally, in a Phase II study with multiple myeloma patients, NOX-A12 combined with bortezomib and dexamethasone was evaluated [137]. Both studies were terminated with unknown results. Recently, an open-label Phase I/II study to evaluate NOX-A12 has started [138]. This study will assess the treatment efficacy of NOX-A12 combined with pembrolizumab in colorectal and pancreatic cancer patients.

## 5. Future Perspectives

The number of available applications of in vitro-selected aptamers has increased significantly over the past 25 years, indicating an exciting future for aptamers as early, personalized cancer diagnostics [139]. Aptamers have demonstrated enormous potential due to their ability to improve previously-established detection methods and their use as novel biosensors for cancer cell imaging and detection [87,107,108]. The simplicity with which aptamers can be modified and conjugated with various molecules (e.g., fluorescent agents, nanoparticles, quantum dots, etc.) will permit their application in various areas of cancer research [140,141]. One such recent application using modified oligonucleotides has made aptamers even more specific and useful [142]. SomaLogic (Boulder, CO, USA) has been generating SOMAmers (slow off-rate modified aptamers) through the incorporation of modified ribose sugars to the aptamer backbone in order to enhance functionality and streamline post-selection optimization [143,144]. The future of aptamer-based cancer diagnosis and biomarker discovery seems to be close to SomaLogic’s development [145]. The creation of new SOMAmer array platforms for targeting more than 1300 different proteins, such as SOMAscan or SOMApanel, suggest that this technology could have a substantial impact in the diagnostic field in the coming years. 

Despite its promising future, the use of aptamers as cancer diagnostics is still mostly restricted to early stages. For several reasons, most currently-available aptamers have not gone beyond the laboratory setting. Cancer cells have complex membrane proteins and vary in composition across both tumor type and subtype, making the accurate identification of tumor cells complex. Further investigation into SELEX techniques are needed to identify high-quality aptamers with improved stability, simplified synthesis, and optimal target specificity. The use of next-generation sequencing (NGS) and bioinformatics coupled with the SELEX method appears to be the critical step needed to address these issues and push aptamers into the clinical setting [19]. In addition to the issues created by the cancer cells themselves, limitations inherent to the aptamers, such as issues with pharmacokinetics, toxicity, and cross-reactivity, remain mostly unresolved and must be addressed before clinical application [146]. Aptamers must be tested under physiologic conditions to validate pharmacokinetic and off-target reactions, and to test for potential changes in toxicity after each modification made to the aptamer [147]. Finally, several authors agree that more standardization and information about aptamers needs to be published, so that more studies of in vitro-selected oligonucleotides can be independently repeated by others [139,148]. The scientific community is being encouraged to publish—at minimum—the sequence information, characterization (*K_d_* values), specificity (cross-reactivity data), secondary structure predictions and the biological, chemical, or thermal stability (buffer conditions, temperature) of their aptamers. This would allow others to reproduce their results—a mechanism critical to the improvement of aptamer-based assays.

## 6. Conclusions

It is important to not only continue with further research and the optimization of existing aptamers, but also to focus on the development of new aptamers with new molecular targets. It is clear that the full potential of aptamer technology has not been reached. Addressing the challenges presented above is crucial to their successful future in personalized medicine. Therefore, the aptamer-based technologies discussed in this review provide an excellent alternative to traditional cancer diagnostic methods, and have the potential to soon radically change how we diagnose and treat cancer.

## Figures and Tables

**Table 1:** A list of reviewed aptamers.

Aptamer Name	Nature	Ligand/Target	Cancer	Affinity (nM) ^1^	Reference in the Review
TD05	DNA	Immunoglobulin heavy constant mu (IGHM)	Burkitt Lymphoma	74.7	[33,75,81,115]
TE02	DNA	Ramos cells	Burkitt Lymphoma	0.76	[33,75]
AS1411	DNA	Nucleolin	Expressed in different cancers	169	[77,99,100,101,108,109,110,120,125]
TLS11a	DNA	HepG2 cells (also LH86 cells)	Hepatocellular carcinoma	7	[78,112,124]
Sgc8	DNA	Tyrosine-protein kinase-like 7 (PTK7)	T-cell leukemia	0.8	[79,80,81,85,92,93,94,97,106]
41t	DNA	Platelet-derived growth factor (PDGF)	---	1	[80]
TE17	DNA	CCRF-CEM cells	T-cell Acute Lymphoblastic Leukemia	675	[80]
KDED 2a-3	DNA	DLD-1 cells	Colorectal cancer	29.2	[81]
KCHA10	DNA	HCT 116 cells	Colorectal cancer	21.3	[81]
Sgd5	DNA	Toledo cells	nonHodgkin’s B cell lymphoma	70.8	[93]
TTA1	DNA	Tenacin C	Expressed in different cancers	5	[99,101]
MUC-1	DNA	Mucin 1	Adenocarcinoma	27	[99]
A32	DNA	Epidermal growth factor receptor III (EGFRIII)	Glioma	0.62	[104]
S11e	DNA	A549 cells	Lung	46.2	[105]
---	RNA	CD30	Lymphoma	0.11	[114]
S6	DNA	A549 cells	Lung	28.2	[116]
J3	DNA	LoVo cells	Colorectal carcinoma	138.2	[117]
F3B	RNA	Human matrix metalloprotease-9 (hMMP-9)	Malignant melanoma	20	[118]
A10	RNA	Prostate membrane antigen (PSMA)	Prostate	11.9	[121,122]
VEGF165	RNA	Vascular endothelial growth factor 165 (VEGF-165)	Expressed in different cancers	50	[123]

^1^ Affinity value using reported dissociation constant (*K_d_*) of each aptamer in nanomoles (nM).
